# *trans*-Dichloridobis[tris­(4-meth­oxy­lphen­yl)phosphane-κ*P*]platinum(II) acetone disolvate

**DOI:** 10.1107/S1600536812045643

**Published:** 2012-11-14

**Authors:** Alfred Muller

**Affiliations:** a Research Centre for Synthesis and Catalysis, Department of Chemistry, University of Johannesburg (APK Campus), PO Box 524, Auckland Park, Johannesburg, 2006, South Africa

## Abstract

In the title compound, [PtCl_2_(C_21_H_21_O_3_P)_2_]·2C_3_H_6_O, the asymmetric unit contains a Pt^II^ ion situated on an inversion center, one chloride anion, one tris­(4-meth­oxy­lphen­yl)phosphane (*L*) ligand and one acetone solvent mol­ecule. The Pt^II^ ion is coordinated by two P atoms [Pt—P = 2.3196 (5) Å] from two *L* ligands and two chloride anions [Pt—Cl = 2.3075 (5) Å] in a distorted square-planar geometry with P—Pt—Cl angles of 88.016 (16) and 91.984 (16)°. The effective cone angle of the phosphane ligand was calculated to be 156°. Weak C—H⋯O and C—H⋯Cl hydrogen bonds hold mol­ecules together.

## Related literature

For related compounds, see: Spessard & Miessler (1996 ▶); van Blerk & Holzapfel (2009 ▶); Muller & Meijboom (2010 ▶). For background to cone angles, see: Tolman (1977 ▶); Otto (2001 ▶).
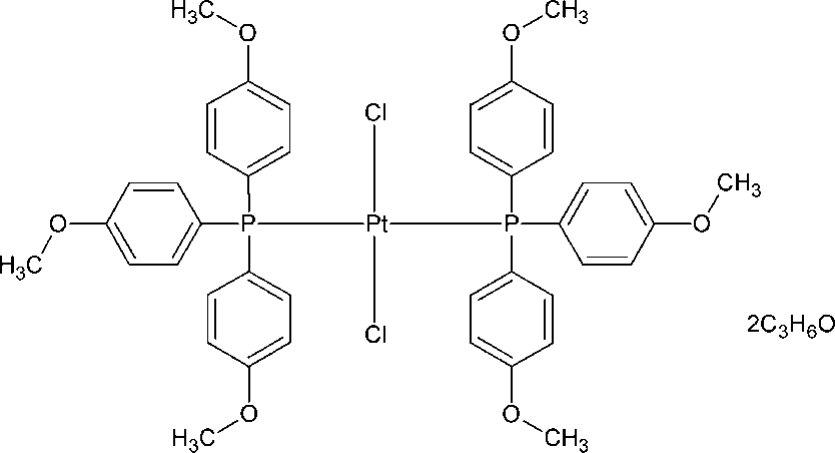


## Experimental

### 

#### Crystal data

[PtCl_2_(C_21_H_21_O_3_P)_2_]·2C_3_H_6_O
*M* *_r_* = 1086.84
Triclinic, 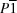

*a* = 10.486 (1) Å
*b* = 11.0360 (11) Å
*c* = 11.3630 (11) Å
α = 85.787 (2)°
β = 63.924 (2)°
γ = 78.370 (2)°
*V* = 1156.70 (19) Å^3^
*Z* = 1
Mo *K*α radiation
μ = 3.27 mm^−1^
*T* = 100 K
0.19 × 0.13 × 0.11 mm

#### Data collection

Bruker APEX DUO 4K CCD diffractometer
Absorption correction: multi-scan (*SADABS*; Bruker, 2008 ▶) *T*_min_ = 0.679, *T*_max_ = 0.746
31470 measured reflections
5799 independent reflections
5763 reflections with *I* > 2σ(*I*)
*R*_int_ = 0.042

#### Refinement

*R*[*F* ^2^ > 2σ(*F* ^2^)] = 0.019
*wR*(*F* ^2^) = 0.040
*S* = 1.04
5799 reflections
282 parameters
H-atom parameters constrained
Δρ_max_ = 0.68 e Å^−3^
Δρ_min_ = −0.51 e Å^−3^

### 

Data collection: *APEX2* (Bruker, 2011 ▶); cell refinement: *SAINT* (Bruker, 2008 ▶); data reduction: *SAINT* and *XPREP* (Bruker, 2008 ▶); program(s) used to solve structure: *SIR97* (Altomare *et al.*, 1999 ▶); program(s) used to refine structure: *SHELXL97* (Sheldrick, 2008 ▶); molecular graphics: *DIAMOND* (Brandenburg & Putz, 2005 ▶); software used to prepare material for publication: *publCIF* (Westrip, 2010 ▶) and *WinGX* (Farrugia, 2012 ▶).

## Supplementary Material

Crystal structure: contains datablock(s) global, I. DOI: 10.1107/S1600536812045643/cv5352sup1.cif

Structure factors: contains datablock(s) I. DOI: 10.1107/S1600536812045643/cv5352Isup2.hkl

Additional supplementary materials:  crystallographic information; 3D view; checkCIF report

## Figures and Tables

**Table 1:** Hydrogen-bond geometry (Å, °)

*D*—H⋯*A*	*D*—H	H⋯*A*	*D*⋯*A*	*D*—H⋯*A*
C6—H6⋯Cl1	0.95	2.7	3.493 (2)	142
C10—H10⋯O1*S* ^i^	0.95	2.57	3.235 (3)	127
C12—H12⋯O2^ii^	0.95	2.53	3.357 (2)	146

Symmetry codes: (i) 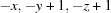; (ii) 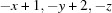.

**Fig. 1.**
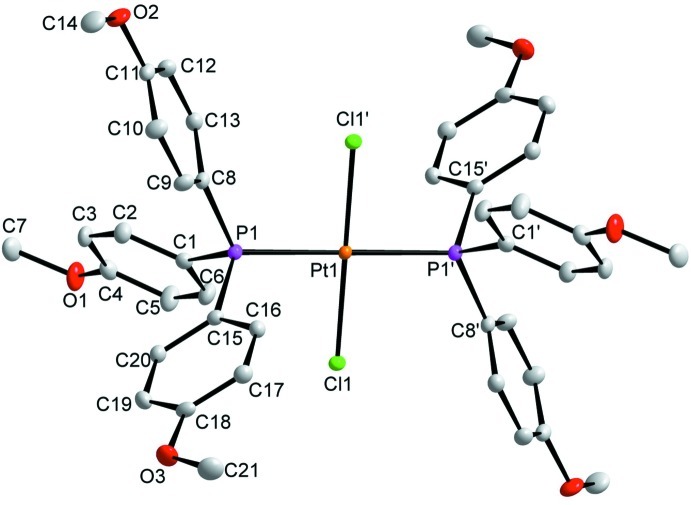
A view of the title complex, showing the atom-numbering scheme and 50% probability displacement ellipsoids [symmetry code: (') 1 - x, 1 - y, 1 - z]. Hydrogen atoms and solvent molecules were omitted for clarity.

## supplementary crystallographic information

### Comment

Transition metal complexes containing phosphane, arsine and stibine ligands are widely being investigated in various fields of organometallic chemistry (Spessard & Miessler, 1996). As part of a systematic investigation (Muller & Meijboom, 2010; van Blerk & Holzapfel, 2009) involving complexes with the general formula *trans/cis*-[*MX*_2_(*L*)_2_] (*M* = Pt or Pd; *X* = halogen, Me, Ph; *L* = Group 15 donor ligand), crystals of the title compound were obtained by the substitution of 1,5-cyclooctadiene (COD) with the tris(4-methoxyphenyl)phosphane from *cis*-[PtCl_2_(COD)].

Molecules of the title compound (Fig. 1) crystallizes in the *P*1 (*Z* = 1) space group with the Pt atom on an inversion center and two acetone solvate molecules accompanying it. Each pair of equivalent ligands is in a *trans* orientation with only slight distortion observed in the P—Pt—Cl angles from the ideal square-planar geometry. The orientation of the phosphanes is such that there is one 4-methoxyphenyl substituent close to the coordination plane, *i.e.* Cl1—Pt1—P1—C8 = -14.92 (7)°. The steric demand of phosphane ligand can be described by using an adaptation of the Tolman cone angle model (Tolman, 1977). By adjusting the Pt—P bond distance to 2.28 Å, and using the geometry from the title compound, an effective cone angle value (Otto, 2001) of 156° was obtained. The packing of the title compound shows weak C—H···Cl/O interactions (Table 1).

### Experimental

*trans*-Dichloridobis[tris(4-methoxylphenyl)phosphane]platinum(II) was prepared from reaction of *cis*-[PtCl_2_(1,5-cyclooctadiene)] (10.8 mg, 0.0289 mmol) and tris(4-methoxyphenyl)phosphane (10 mg, 0.0259 mmol) in acetone. The mixture was refluxed for 4hrs, then filtered and crystals suitable for a single-crystal X-ray diffraction study was obtained by recrystallization from acetone. Analytical data: ^31^P {H} NMR (CDCl_3_, 161.99 MHz): δ = 16.4 (t, ^1^*J*(^31^P-^195^Pt) = 2586 Hz).

### Refinement

The aromatic and methyl H atoms were placed in geometrically idealized positions (C—H = 0.95–0.98) and allowed to ride on their parent atoms, with *U*_iso_(H) = 1.2*U*_eq_(C) for aromatic and *U*_iso_(H) = 1.5*U*_eq_(C) for methyl H atoms respectively. Methyl torsion angles were refined from electron density. Residual electron density values (< 1 Å^-3^) are within 1 Å from Pt and represent no physical meaning.

### Crystal data

**Table:**

[PtCl_2_(C_21_H_21_O_3_P)_2_]·2C_3_H_6_O	*Z* = 1
*M**_r_* = 1086.84	*F*(000) = 548
Triclinic, *P*1	*D*_x_ = 1.56 Mg m^−^^3^
*a* = 10.486 (1) Å	Mo *K*α radiation, λ = 0.71069 Å
*b* = 11.0360 (11) Å	Cell parameters from 9903 reflections
*c* = 11.3630 (11) Å	θ = 2.2–32.7°
α = 85.787 (2)°	µ = 3.27 mm^−^^1^
β = 63.924 (2)°	*T* = 100 K
γ = 78.370 (2)°	Rectangle, colourless
*V* = 1156.70 (19) Å^3^	0.19 × 0.13 × 0.11 mm

### Data collection

**Table:**

Bruker APEX DUO 4K CCD diffractometer	5799 independent reflections
Graphite monochromator	5763 reflections with *I* > 2σ(*I*)
Detector resolution: 8.4 pixels mm^-1^	*R*_int_ = 0.042
φ and ω scans	θ_max_ = 28.4°, θ_min_ = 1.9°
Absorption correction: multi-scan (*SADABS*; Bruker, 2008)	*h* = −13→14
*T*_min_ = 0.679, *T*_max_ = 0.746	*k* = −14→14
31470 measured reflections	*l* = −15→15

### Refinement

**Table:**

Refinement on *F*^2^	Primary atom site location: structure-invariant direct methods
Least-squares matrix: full	Secondary atom site location: difference Fourier map
*R*[*F*^2^ > 2σ(*F*^2^)] = 0.019	Hydrogen site location: inferred from neighbouring sites
*wR*(*F*^2^) = 0.040	H-atom parameters constrained
*S* = 1.04	*w* = 1/[σ^2^(*F*_o_^2^) + (0.016*P*)^2^ + 0.4415*P*] where *P* = (*F*_o_^2^ + 2*F*_c_^2^)/3
5799 reflections	(Δ/σ)_max_ = 0.001
282 parameters	Δρ_max_ = 0.68 e Å^−^^3^
0 restraints	Δρ_min_ = −0.51 e Å^−^^3^

### Special details

**Table:**

Experimental. The intensity data was collected on a Bruker Apex DUO 4 K CCD diffractometer using an exposure time of 20 s/frame. A total of 2352 frames were collected with a frame width of 0.5° covering up to θ = 28.66° with 99.3% completeness accomplished.
Geometry. All e.s.d.'s (except the e.s.d. in the dihedral angle between two l.s. planes) are estimated using the full covariance matrix. The cell e.s.d.'s are taken into account individually in the estimation of e.s.d.'s in distances, angles and torsion angles; correlations between e.s.d.'s in cell parameters are only used when they are defined by crystal symmetry. An approximate (isotropic) treatment of cell e.s.d.'s is used for estimating e.s.d.'s involving l.s. planes.
Refinement. Refinement of *F*^2^ against ALL reflections. The weighted *R*-factor *wR* and goodness of fit *S* are based on *F*^2^, conventional *R*-factors *R* are based on *F*, with *F* set to zero for negative *F*^2^. The threshold expression of *F*^2^ > σ(*F*^2^) is used only for calculating *R*-factors(gt) *etc*. and is not relevant to the choice of reflections for refinement. *R*-factors based on *F*^2^ are statistically about twice as large as those based on *F*, and *R*- factors based on ALL data will be even larger.

### Fractional atomic coordinates and isotropic or equivalent isotropic displacement parameters (Å^2^)

**Table:**

	*x*	*y*	*z*	*U*_iso_*/*U*_eq_	
Pt1	0.5	0.5	0.5	0.00927 (3)	
Cl1	0.52266 (5)	0.28766 (4)	0.49934 (4)	0.01424 (9)	
P1	0.49557 (5)	0.49994 (4)	0.29769 (5)	0.01069 (9)	
O2	0.31108 (15)	0.97824 (13)	0.08244 (15)	0.0210 (3)	
O3	0.06877 (15)	0.21272 (14)	0.30254 (14)	0.0212 (3)	
O1	1.08770 (15)	0.32059 (15)	−0.12406 (14)	0.0225 (3)	
O1S	0.08106 (17)	0.10043 (16)	0.63614 (17)	0.0353 (4)	
C20	0.4006 (2)	0.32915 (18)	0.19434 (19)	0.0146 (4)	
H20	0.4908	0.3216	0.1189	0.017*	
C6	0.7760 (2)	0.35873 (19)	0.1910 (2)	0.0190 (4)	
H6	0.7519	0.3277	0.2769	0.023*	
C19	0.2972 (2)	0.26563 (18)	0.1987 (2)	0.0174 (4)	
H19	0.3177	0.2136	0.1272	0.021*	
C18	0.1634 (2)	0.27795 (18)	0.3078 (2)	0.0153 (4)	
C5	0.9129 (2)	0.3196 (2)	0.0926 (2)	0.0220 (5)	
H5	0.9821	0.2622	0.1112	0.026*	
C17	0.1346 (2)	0.35162 (18)	0.4140 (2)	0.0160 (4)	
H17	0.0441	0.3598	0.4891	0.019*	
C1	0.67224 (19)	0.44348 (17)	0.16598 (18)	0.0128 (4)	
C8	0.43388 (19)	0.64708 (17)	0.23846 (18)	0.0123 (4)	
C3	0.8488 (2)	0.44858 (19)	−0.06024 (19)	0.0165 (4)	
H3	0.8737	0.4804	−0.1459	0.02*	
C15	0.37324 (19)	0.40419 (17)	0.29972 (18)	0.0124 (4)	
C12	0.4759 (2)	0.84717 (19)	0.14382 (19)	0.0171 (4)	
H12	0.5356	0.9077	0.1135	0.02*	
C11	0.3432 (2)	0.86913 (18)	0.13752 (19)	0.0149 (4)	
C16	0.2402 (2)	0.41291 (18)	0.40867 (19)	0.0141 (4)	
H16	0.221	0.4624	0.4817	0.017*	
C10	0.2547 (2)	0.78264 (19)	0.1844 (2)	0.0183 (4)	
H10	0.1631	0.7982	0.1827	0.022*	
C7	1.1264 (2)	0.3620 (2)	−0.2550 (2)	0.0215 (4)	
H7A	1.0643	0.3361	−0.2886	0.032*	
H7B	1.2276	0.326	−0.3104	0.032*	
H7C	1.1137	0.4525	−0.2561	0.032*	
C4	0.9497 (2)	0.36409 (18)	−0.03364 (19)	0.0158 (4)	
C1S	0.2096 (2)	0.0584 (2)	0.5897 (2)	0.0253 (5)	
C9	0.3009 (2)	0.67246 (18)	0.2342 (2)	0.0164 (4)	
H9	0.2397	0.613	0.2661	0.02*	
C14	0.1812 (2)	0.9985 (2)	0.0649 (2)	0.0245 (5)	
H14A	0.1845	0.9306	0.0121	0.037*	
H14B	0.1726	1.0769	0.0201	0.037*	
H14C	0.0975	1.002	0.1507	0.037*	
C2	0.7115 (2)	0.48623 (19)	0.03891 (19)	0.0165 (4)	
H2	0.6423	0.5428	0.0196	0.02*	
C13	0.5205 (2)	0.73802 (18)	0.19384 (19)	0.0151 (4)	
H13	0.6107	0.7241	0.1982	0.018*	
C21	−0.0678 (2)	0.2190 (2)	0.4155 (2)	0.0253 (5)	
H21A	−0.0516	0.1871	0.4915	0.038*	
H21B	−0.126	0.169	0.3992	0.038*	
H21C	−0.119	0.3052	0.4327	0.038*	
C2S	0.2949 (3)	0.0285 (2)	0.4466 (2)	0.0388 (7)	
H2S1	0.2289	0.0343	0.4057	0.058*	
H2S2	0.3517	−0.0557	0.4346	0.058*	
H2S3	0.3602	0.0873	0.4056	0.058*	
C3S	0.2896 (3)	0.0339 (2)	0.6725 (3)	0.0385 (6)	
H3S1	0.2234	0.0597	0.7632	0.058*	
H3S2	0.3685	0.0805	0.6393	0.058*	
H3S3	0.3295	−0.0548	0.6693	0.058*	

### Atomic displacement parameters (Å^2^)

**Table:**

	*U*^11^	*U*^22^	*U*^33^	*U*^12^	*U*^13^	*U*^23^
Pt1	0.00961 (5)	0.00951 (5)	0.00805 (5)	−0.00186 (3)	−0.00334 (4)	0.00087 (4)
Cl1	0.0185 (2)	0.0105 (2)	0.0130 (2)	−0.00282 (17)	−0.00628 (18)	0.00104 (17)
P1	0.0110 (2)	0.0113 (2)	0.0095 (2)	−0.00231 (18)	−0.00417 (18)	0.00079 (18)
O2	0.0229 (7)	0.0151 (7)	0.0244 (8)	−0.0027 (6)	−0.0113 (6)	0.0078 (6)
O3	0.0204 (7)	0.0245 (8)	0.0227 (8)	−0.0114 (6)	−0.0098 (6)	−0.0001 (6)
O1	0.0129 (7)	0.0352 (9)	0.0120 (7)	0.0017 (6)	−0.0010 (6)	−0.0017 (6)
O1S	0.0215 (8)	0.0367 (10)	0.0382 (10)	−0.0019 (7)	−0.0049 (8)	−0.0059 (8)
C20	0.0154 (9)	0.0151 (10)	0.0121 (9)	−0.0033 (7)	−0.0050 (8)	0.0009 (7)
C6	0.0204 (10)	0.0200 (11)	0.0131 (10)	0.0004 (8)	−0.0062 (8)	0.0029 (8)
C19	0.0227 (10)	0.0158 (10)	0.0157 (10)	−0.0039 (8)	−0.0097 (8)	−0.0013 (8)
C18	0.0174 (9)	0.0133 (9)	0.0196 (10)	−0.0060 (7)	−0.0113 (8)	0.0048 (8)
C5	0.0180 (10)	0.0261 (12)	0.0160 (10)	0.0057 (8)	−0.0062 (8)	0.0004 (9)
C17	0.0145 (9)	0.0168 (10)	0.0165 (10)	−0.0039 (7)	−0.0065 (8)	0.0018 (8)
C1	0.0114 (8)	0.0132 (9)	0.0135 (9)	−0.0038 (7)	−0.0043 (7)	−0.0004 (7)
C8	0.0136 (9)	0.0129 (9)	0.0097 (9)	−0.0014 (7)	−0.0048 (7)	0.0007 (7)
C3	0.0174 (9)	0.0199 (10)	0.0092 (9)	−0.0028 (8)	−0.0034 (8)	0.0004 (8)
C15	0.0131 (8)	0.0116 (9)	0.0133 (9)	−0.0029 (7)	−0.0066 (7)	0.0024 (7)
C12	0.0192 (10)	0.0166 (10)	0.0160 (10)	−0.0074 (8)	−0.0070 (8)	0.0026 (8)
C11	0.0179 (9)	0.0131 (9)	0.0112 (9)	−0.0006 (7)	−0.0050 (8)	0.0004 (7)
C16	0.0160 (9)	0.0140 (9)	0.0125 (9)	−0.0027 (7)	−0.0062 (8)	−0.0002 (7)
C10	0.0152 (9)	0.0177 (10)	0.0228 (11)	−0.0028 (8)	−0.0098 (8)	0.0040 (8)
C7	0.0166 (10)	0.0268 (12)	0.0142 (10)	−0.0046 (8)	0.0001 (8)	−0.0028 (9)
C4	0.0121 (9)	0.0187 (10)	0.0141 (10)	−0.0024 (7)	−0.0030 (8)	−0.0037 (8)
C1S	0.0250 (11)	0.0141 (10)	0.0307 (13)	−0.0049 (9)	−0.0064 (10)	0.0028 (9)
C9	0.0158 (9)	0.0147 (10)	0.0182 (10)	−0.0055 (8)	−0.0065 (8)	0.0043 (8)
C14	0.0261 (11)	0.0195 (11)	0.0297 (12)	0.0009 (9)	−0.0167 (10)	0.0052 (9)
C2	0.0147 (9)	0.0192 (10)	0.0144 (10)	−0.0003 (8)	−0.0064 (8)	0.0008 (8)
C13	0.0156 (9)	0.0166 (10)	0.0133 (9)	−0.0043 (8)	−0.0060 (8)	0.0003 (8)
C21	0.0204 (10)	0.0275 (12)	0.0306 (13)	−0.0112 (9)	−0.0108 (10)	0.0027 (10)
C2S	0.0381 (14)	0.0278 (14)	0.0300 (14)	0.0102 (11)	−0.0042 (11)	0.0084 (11)
C3S	0.0387 (14)	0.0326 (14)	0.0456 (16)	−0.0033 (11)	−0.0208 (13)	−0.0010 (12)

### Geometric parameters (Å, º)

**Table:**

Pt1—Cl1^i^	2.3075 (5)	C3—C4	1.386 (3)
Pt1—Cl1	2.3075 (5)	C3—H3	0.95
Pt1—P1	2.3196 (5)	C15—C16	1.390 (3)
Pt1—P1^i^	2.3197 (5)	C12—C13	1.378 (3)
P1—C15	1.8110 (19)	C12—C11	1.396 (3)
P1—C1	1.8151 (19)	C12—H12	0.95
P1—C8	1.8185 (19)	C11—C10	1.380 (3)
O2—C11	1.366 (2)	C16—H16	0.95
O2—C14	1.433 (2)	C10—C9	1.391 (3)
O3—C18	1.361 (2)	C10—H10	0.95
O3—C21	1.435 (3)	C7—H7A	0.98
O1—C4	1.366 (2)	C7—H7B	0.98
O1—C7	1.426 (2)	C7—H7C	0.98
O1S—C1S	1.212 (3)	C1S—C3S	1.492 (3)
C20—C19	1.386 (3)	C1S—C2S	1.495 (3)
C20—C15	1.397 (3)	C9—H9	0.95
C20—H20	0.95	C14—H14A	0.98
C6—C5	1.381 (3)	C14—H14B	0.98
C6—C1	1.401 (3)	C14—H14C	0.98
C6—H6	0.95	C2—H2	0.95
C19—C18	1.394 (3)	C13—H13	0.95
C19—H19	0.95	C21—H21A	0.98
C18—C17	1.391 (3)	C21—H21B	0.98
C5—C4	1.390 (3)	C21—H21C	0.98
C5—H5	0.95	C2S—H2S1	0.98
C17—C16	1.387 (3)	C2S—H2S2	0.98
C17—H17	0.95	C2S—H2S3	0.98
C1—C2	1.390 (3)	C3S—H3S1	0.98
C8—C9	1.388 (3)	C3S—H3S2	0.98
C8—C13	1.406 (3)	C3S—H3S3	0.98
C3—C2	1.384 (3)		
			
Cl1^i^—Pt1—Cl1	180.0000 (10)	C17—C16—C15	122.03 (18)
Cl1^i^—Pt1—P1	91.984 (16)	C17—C16—H16	119
Cl1—Pt1—P1	88.016 (16)	C15—C16—H16	119
Cl1^i^—Pt1—P1^i^	88.016 (16)	C11—C10—C9	119.42 (18)
Cl1—Pt1—P1^i^	91.984 (16)	C11—C10—H10	120.3
P1—Pt1—P1^i^	180	C9—C10—H10	120.3
C15—P1—C1	107.86 (9)	O1—C7—H7A	109.5
C15—P1—C8	103.27 (8)	O1—C7—H7B	109.5
C1—P1—C8	103.77 (9)	H7A—C7—H7B	109.5
C15—P1—Pt1	110.80 (6)	O1—C7—H7C	109.5
C1—P1—Pt1	112.70 (6)	H7A—C7—H7C	109.5
C8—P1—Pt1	117.60 (6)	H7B—C7—H7C	109.5
C11—O2—C14	116.83 (16)	O1—C4—C3	124.33 (18)
C18—O3—C21	117.38 (16)	O1—C4—C5	115.86 (17)
C4—O1—C7	116.81 (15)	C3—C4—C5	119.80 (18)
C19—C20—C15	120.62 (18)	O1S—C1S—C3S	121.7 (2)
C19—C20—H20	119.7	O1S—C1S—C2S	121.3 (2)
C15—C20—H20	119.7	C3S—C1S—C2S	117.0 (2)
C5—C6—C1	120.95 (18)	C8—C9—C10	121.71 (18)
C5—C6—H6	119.5	C8—C9—H9	119.1
C1—C6—H6	119.5	C10—C9—H9	119.1
C20—C19—C18	120.17 (18)	O2—C14—H14A	109.5
C20—C19—H19	119.9	O2—C14—H14B	109.5
C18—C19—H19	119.9	H14A—C14—H14B	109.5
O3—C18—C17	124.15 (18)	O2—C14—H14C	109.5
O3—C18—C19	115.85 (18)	H14A—C14—H14C	109.5
C17—C18—C19	119.99 (18)	H14B—C14—H14C	109.5
C6—C5—C4	120.14 (18)	C3—C2—C1	121.79 (17)
C6—C5—H5	119.9	C3—C2—H2	119.1
C4—C5—H5	119.9	C1—C2—H2	119.1
C16—C17—C18	118.99 (18)	C12—C13—C8	120.71 (17)
C16—C17—H17	120.5	C12—C13—H13	119.6
C18—C17—H17	120.5	C8—C13—H13	119.6
C2—C1—C6	117.78 (17)	O3—C21—H21A	109.5
C2—C1—P1	121.60 (14)	O3—C21—H21B	109.5
C6—C1—P1	120.56 (15)	H21A—C21—H21B	109.5
C9—C8—C13	117.93 (17)	O3—C21—H21C	109.5
C9—C8—P1	121.10 (14)	H21A—C21—H21C	109.5
C13—C8—P1	120.97 (14)	H21B—C21—H21C	109.5
C2—C3—C4	119.52 (18)	C1S—C2S—H2S1	109.5
C2—C3—H3	120.2	C1S—C2S—H2S2	109.5
C4—C3—H3	120.2	H2S1—C2S—H2S2	109.5
C16—C15—C20	118.19 (17)	C1S—C2S—H2S3	109.5
C16—C15—P1	117.96 (14)	H2S1—C2S—H2S3	109.5
C20—C15—P1	123.68 (15)	H2S2—C2S—H2S3	109.5
C13—C12—C11	120.23 (18)	C1S—C3S—H3S1	109.5
C13—C12—H12	119.9	C1S—C3S—H3S2	109.5
C11—C12—H12	119.9	H3S1—C3S—H3S2	109.5
O2—C11—C10	124.34 (17)	C1S—C3S—H3S3	109.5
O2—C11—C12	115.70 (17)	H3S1—C3S—H3S3	109.5
C10—C11—C12	119.96 (18)	H3S2—C3S—H3S3	109.5
			
Cl1^i^—Pt1—P1—C15	−133.32 (7)	C1—P1—C15—C16	166.68 (14)
Cl1—Pt1—P1—C15	46.68 (7)	C8—P1—C15—C16	−83.88 (16)
Cl1^i^—Pt1—P1—C1	105.72 (7)	Pt1—P1—C15—C16	42.91 (16)
Cl1—Pt1—P1—C1	−74.28 (7)	C1—P1—C15—C20	−18.18 (18)
Cl1^i^—Pt1—P1—C8	−14.92 (7)	C8—P1—C15—C20	91.26 (17)
Cl1—Pt1—P1—C8	165.08 (7)	Pt1—P1—C15—C20	−141.95 (14)
C15—C20—C19—C18	1.2 (3)	C14—O2—C11—C10	−4.5 (3)
C21—O3—C18—C17	−1.4 (3)	C14—O2—C11—C12	175.03 (18)
C21—O3—C18—C19	177.40 (17)	C13—C12—C11—O2	−177.95 (18)
C20—C19—C18—O3	179.52 (17)	C13—C12—C11—C10	1.6 (3)
C20—C19—C18—C17	−1.6 (3)	C18—C17—C16—C15	0.8 (3)
C1—C6—C5—C4	−0.1 (3)	C20—C15—C16—C17	−1.2 (3)
O3—C18—C17—C16	179.38 (17)	P1—C15—C16—C17	174.19 (15)
C19—C18—C17—C16	0.6 (3)	O2—C11—C10—C9	177.62 (19)
C5—C6—C1—C2	0.3 (3)	C12—C11—C10—C9	−1.8 (3)
C5—C6—C1—P1	−176.99 (17)	C7—O1—C4—C3	−2.8 (3)
C15—P1—C1—C2	88.16 (17)	C7—O1—C4—C5	178.13 (18)
C8—P1—C1—C2	−20.94 (18)	C2—C3—C4—O1	179.84 (19)
Pt1—P1—C1—C2	−149.22 (14)	C2—C3—C4—C5	−1.1 (3)
C15—P1—C1—C6	−94.64 (17)	C6—C5—C4—O1	179.66 (19)
C8—P1—C1—C6	156.27 (16)	C6—C5—C4—C3	0.5 (3)
Pt1—P1—C1—C6	27.99 (18)	C13—C8—C9—C10	1.7 (3)
C15—P1—C8—C9	14.06 (18)	P1—C8—C9—C10	−177.47 (16)
C1—P1—C8—C9	126.52 (16)	C11—C10—C9—C8	0.2 (3)
Pt1—P1—C8—C9	−108.28 (16)	C4—C3—C2—C1	1.3 (3)
C15—P1—C8—C13	−165.06 (16)	C6—C1—C2—C3	−0.9 (3)
C1—P1—C8—C13	−52.59 (17)	P1—C1—C2—C3	176.36 (16)
Pt1—P1—C8—C13	72.60 (17)	C11—C12—C13—C8	0.4 (3)
C19—C20—C15—C16	0.2 (3)	C9—C8—C13—C12	−2.0 (3)
C19—C20—C15—P1	−174.93 (15)	P1—C8—C13—C12	177.19 (15)

Symmetry code: (i) −*x*+1, −*y*+1, −*z*+1.

### Hydrogen-bond geometry (Å, º)

**Table:**

*D*—H···*A*	*D*—H	H···*A*	*D*···*A*	*D*—H···*A*
C6—H6···Cl1	0.95	2.7	3.493 (2)	142
C10—H10···O1*S*^ii^	0.95	2.57	3.235 (3)	127
C12—H12···O2^iii^	0.95	2.53	3.357 (2)	146

Symmetry codes: (ii) −*x*, −*y*+1, −*z*+1; (iii) −*x*+1, −*y*+2, −*z*.
